# Patients' experiences of the caring encounter with the psychiatric emergency response team in the emergency medical service—A qualitative interview study

**DOI:** 10.1111/hex.13024

**Published:** 2020-01-22

**Authors:** Veronica Lindström, Lars Sturesson, Andreas Carlborg

**Affiliations:** ^1^ Academic EMS Stockholm Sweden; ^2^ Department of Neurobiology Karolinska Institutet Care Sciences, and Society Division of Nursing Stockholm Stockholm Sweden; ^3^ Department of Clinical Neuroscience Centre for psychiatric research Karolinska Institutet Stockholm Sweden

**Keywords:** ambulance, emergency medical service, mental illness, patient experience, psychiatric emergency response team, suicide prevention

## Abstract

**Background:**

Mental illnesses are increasing in the population; consequently, the number of psychiatric emergencies handled by the emergency medical services (EMS) has also increased. Alternative response systems have been developed and evaluated, but there is still a lack of knowledge concerning the patients' experiences of being cared for in the EMS by a psychiatric emergency response unit (In Swedish: Psykiatrisk Akut Mobilitet [PAM]).

**Objective:**

The aim of this study was to explore patients' experiences of the caring encounter with the PAM team.

**Design:**

A qualitative study design with 14 patients' interviews and content analysis was used.

**Results:**

The patients expressed that the PAM team created a safe environment and actively involved the patient in their care by creating an open and safe place for dialogue. In this safe environment, the patients described how they participated in the decision making and received care without fear of being dismissed, ignored or judged.

**Discussion and Conclusion:**

The patients' experiences of being cared for by the PAM team show that person‐centred care was achieved by involving the patients in their own care. This participation was possible because mutual trust and confidence existed, and the patients acknowledged the specialist response unit to be a valuable part of the EMS. However, further studies are needed to explore whether the PAM as a response unit in the EMS decreases the risk of suicide and to examine different health economic aspects of using PAM in the EMS.

## 1 BACKGROUND

Mental illnesses are today regarded as a public health problem and are increasing in the population, especially among young adults and older people.^1^, ^2^ Consequently, the number of psychiatric emergencies handled by the emergency medical services (EMS) has also increased.^3^ Handling psychiatric emergencies such as mental health crises is both challenging and complex for EMS personnel due to their insufficient education^4^, ^5^ and limited direct access to psychiatric specialist services.^6^ Accordingly, alternative response systems for psychiatric emergencies have been developed and evaluated over the years, and the use of specialized psychiatric response organizations as part of law enforcement and/or the EMS has been shown to be positive overall from the perspective of the personnel and the organizations.^7^, ^8^, ^9^, ^10^, ^11^, ^12^, ^13^, ^14^ Previous studies have also shown that an early response unit with special knowledge of handling patients in psychiatric emergency situations could help the patient to achieve a preferred outcome.^7^ However, there is a lack of knowledge concerning the patients' perspective of the care. From a caring perspective, we need to acquire more knowledge and understanding of the patients' experiences of received care; otherwise, it may be hard to develop and show sensitivity and compassion for these vulnerable patients. With lack of knowledge of patients' experiences, it is also hard to develop care and to conduct caring intervention studies.^15^

According to Holopainen, Nyström & Kasén, ‘A caring encounter arises between two equal persons where one is a nurse and the other is a patient’ p. 14*.* 16 The caring encounter in the EMS has challenges since the environment is unpredictable, the encounter may not always be on equal terms, and in addition the encounter sometimes takes place in public environment. The EMS personnel therefore need to be open or prepared and to take responsibility for the caring encounter,^17^ and the encounter depends on the professional‐patient relation and communication.^18^ To retain his/her identity by means of communicative contact, the patient also needs to be confirmed in the encounter.^19^ At present, there is a lack of knowledge about how patients with mental illness experience the caring encounter in the EMS.

In Sweden, where this study was conducted, mental illness is increasing and there are several ways to access mental health‐care services, including via general practitioners, community mental health services, psychiatric outreach teams, psychiatric emergency departments (PED), telephone services and private health practitioners. However, in a psychiatric emergency situation, that is a threatening suicide attempt, it is usually the police, rescue department and/or the EMS, including the ambulance service, who respond. Since 2015, there has also been a psychiatric emergency response team (In Swedish: Psykiatrisk Akut Mobilitet [PAM]) operating in the county of Stockholm in Sweden which responds to psychiatric emergencies.^9^

### 1.1 The psychiatric emergency response team

The main purpose of the PAM is to respond to emergency calls regarding persons (of all ages) facing acute crises or acute mental illness, with suicide prevention as the main priority. The vehicle used resembles an ordinary ambulance and is equipped with a blue light and sirens, computers for mobile access to patients' medical records and medications such as basic tranquilizers, sleep medication and antipsychotic drugs. The interior of the vehicle consists of four rotating seats allowing both assessment and transport of the patient. The PAM is operative between 14:00 and 02:00 daily and is manned by two registered nurses (RNs) specialized in psychiatric care and one emergency medical technician (EMT). The RNs have one year of additional training at university level and hold master's degrees. The EMTs have education in and knowledge of basic life support and radio communication, and are licensed to drive a vehicle with blue lights and sirens.^9^, ^20^ The emergency medical communication centre (EMCC) dispatches the PAM after assessing the emergency call as a psychiatric emergency situation appropriate for the PAM team to handle. In accordance with the Swedish Medical Index used by the EMCC, the PAM is dispatched according to three levels of priority: (a) ‘acute life‐threatening situation/condition with blue light and sirens’; (b) ‘acute but not life‐threatening’; and (c) ‘transportation to hospital/other caregivers may be needed’.^21^ If necessary, additional resources such as the police, rescue department and/or ambulances are also dispatched by the EMCC. It is common for the PAM team to cooperate with the police, ambulance, rescue department and somatic emergency departments (ED) 9 due to the nature of the illness. A patient in need of further assessment and inpatient care will be transported by the PAM team and admitted to the appropriate ED (psychiatric, somatic or substance‐use ED). The PAM can also decide to pass the assignment to the ordinary ambulance service if the medical condition requires this, or leave the patient at the scene without further action. When the patients are not transported to the next level of care, the PAM team gives advice for self‐care and/or when necessary support in contacting the patient's ordinary caregiver. The RNs also have the opportunity to use nurse‐initiated protocols for basic tranquilizers, sleep medication and antipsychotic drugs. The team also have the option of communicating with a psychiatrist on call in the PED if the team has a need for medical consultation.^9^ The use of the PAM or a similar specialized psychiatric response unit as a part of the EMS or the police department has been evaluated from the organization's perspective, showing that one third of all PAM assignments resulted in no further action after a psychiatric assessment and/or a crisis intervention carried out at the scene.^9^ Nevertheless, there is a knowledge gap concerning the patients' experiences. Therefore, the aim of this study was to explore patients' experiences of the caring encounter with the PAM team. By exploring the patients' perspectives, this study is intended to contribute knowledge about using a psychiatric emergency response team as first responders to care seekers in a psychiatric emergency situation.

## 2 METHODS

### 2.1 Research design

This study has been carried out using a qualitative study design, and interviews were conducted and analysed to explore patients' experiences of the caring encounter with the PAM.

### 2.2 Setting

The study was conducted in the county of Stockholm, which has a population of approximately 2.3 million. The county has one PED, providing a 24‐hour psychiatric emergency service. The PED handles around 22 000 patient consultations yearly. There are also numerous mobile psychiatric teams throughout the county. However, these mobile psychiatric teams usually make scheduled visits and operate between 8 am and 10 pm, and they do not always have the capacity to respond to psychiatric emergency situations such as ongoing suicide attempts. During the first year of operation (April 2015–March 2016), PAM handled 1254 assignments. These assignments, according to the Swedish Medical Index, 21 included treatment of serious suicide attempts (36%), suspicion of severe psychiatric illness (25%), acute crisis (18%), serious suicide attempts (6%), suspicion of intoxication (3%) and undefined problems/difficult to assess (12%). Of these, 40% led to hospital admissions and the majority of patients were female (56%) with an age range between 18 and 29 years (27%). The PAM team collaborated with the ambulance service (55%), the police (49%) and the rescue department (7%) in 76% of the assignments.^9^

### 2.3 Participants and Data collection

The data collection was carried out between March and June 2018 and included a convenience sample of Swedish‐speaking patients who had been cared for by the PAM team. The convenience sampling required that the patients should be able to understand written and oral information about the study and were willing to participate in the study at the end of the caring encounter. In assignments where the PAM team were the first and only responders, the responsible specialist nurse asked the patients to confirm their willingness to participate. Patients' previous experience of the PAM did not affect the question of possible participation in the study. Approximately 7‐10 days after the caring encounter, the researchers contacted the patients by phone and asked if they had read the written information that they had received from the PAM team, understood the aim of the study and were willing to participate in an interview. In total, 30 patients were willing to participate in the study when the specialist nurse from the PAM asked, and of these, 16 patients were reachable (53%). At times and places selected by the participants, one face‐to‐face and 15 telephone semi‐structured interviews were conducted. The participants were when contacted by the researcher proposed to meet face‐to‐face for the interview, but when they denied a face‐to‐face meeting they were proposed a telephone interview instead. Telephone interviews were proposed to extend the access to the participants, despite the known risk of being unable to observe the participants' body language during the interviews.^22^ These interviews lasted from 15 to 45 minutes (mean 20 minutes, not including the informed consent) and were digitally recorded, anonymized and transcribed in their entirety. All the interviews started with a short recorded presentation of the study's aim and confirmation of informed consent. To elicit the patient's recall of the encounter, the interviewer then started with the question ‘Can you tell me what happened and why you met the PAM?’ This question was then supplemented by questions from a semi‐structured interview guide and follow‐up questions such as ‘Can you tell me more…? What did you think about that…? Can you give example? … Can you describe’ (see Table 1). The development of the interview guide was inspired by previous studies concerning patient participation in the emergency setting.^18^, ^19^, ^23^ Data collection continued until no new information was obtained during the interviews, and all patients who were asked to participate in the study were contacted by the researchers.

**Table 1:** The semi‐structured interview guide

How did you experience the caring encounter and treatment by the personnel?
What were your expectations of the care?
Did you receive the help you asked for?
In what way could you influence your care?
In what way did the personnel utilize your previous experiences?
What advice did you get about how to treat symptoms or other problems?
Is there anything else you want to add?

### 2.4 Analysis

A qualitative inductive content analysis 24 and a caring science approach 25 were used to analyse the collected data. The caring science approach entailed use of the authors’ theoretical and clinical knowledge for valuing, interpreting and understanding the collected data. The analysis was carried out in three phases. The first phase, the preparation phase, started with two of the authors transcribing verbatim and reading the entire body of data, with the aim of obtaining an initial understanding of the interviews. Due to lack of a good dialogue and meaningful statements, two of the interviews were excluded in the preparation phase. After the initial reading, the data from 14 interviews were divided into meaningful units describing patients’ experiences of the caring encounter with the PAM team. The second phase, the organization phase, involved clustering the units into codes to identify similarities and discrepancies in the collected data. The codes were then sorted into broader subcategories until patterns were identified as meaningful in relation to the study aim. In the third phase, the reporting phase, the patients’ experiences of the caring encounter with the PAM team, was categorized into three categories and one main category. The different phases were initiated by one of the authors and then discussed to achieve consistency among all authors. In accordance with the exploratory aim of this study, the main category represented general rather than specific descriptions of the patients’ experiences of the caring encounter with the PAM. During the whole analysis, there was a continuous movement between the interviews, codes and subcategories, categories and main category, with the aim of preserving the essence of the patients’ reported experiences. This continuous movement and the discussions among the researchers are awareness techniques to maintain a balance between the researchers’ pre‐understanding and their openness to the content. The researchers’ pre‐understanding in this study included extensive knowledge of the EMS setting, psychiatric medicine and emergency nursing, but there was no relationship between the authors and the participants in this study.

## 3 RESULTS

The majority of participants were female, and nine of the participants were transported by the PAM team to the hospital as shown in Table 2. The exploration of the patients experiences of the caring encounter with the PAM team resulted in three categories and one main category.

**Table 2:** Characteristics of the participants

	n = 14
Age (range)	23‐61
Average age	36
Male	5
Female	9
Non–native Swedish‐speaking	4
Previous experience of PAM	4
Made the call to the EMCC	
Parents	3
Care seeker	6
Next of kin	4
Missing	1
Primary psychiatric assessment	
Anxiety/Self‐harm	5/3
Depression/Suicidal threats	3/2
Other, that is psychosis	1
Admissions	
Assessment by physician at the PED	3
Voluntary inpatient care (constraint care)	5 (1)
Self‐care advice	5

Abbreviations: EMCC, emergency medical communication centre; PAM, psychiatric emergency response unit; PED, psychiatric emergency department.

**Figure 1:**
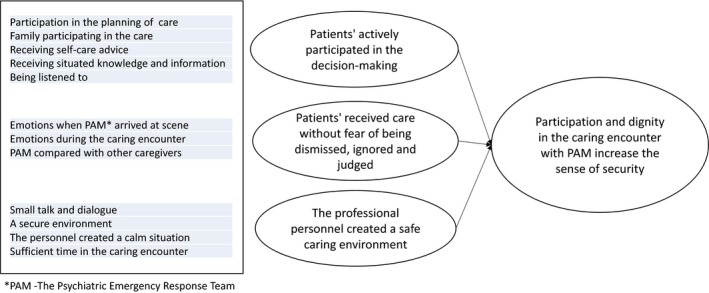
Illustration of the results

The main category, ‘Participation and dignity in the caring encounter with PAM increase the sense of security’ illustrates the importance of creating a safe environment and actively involving the patient in his or her care by creating an open and safe place for dialogue. In this safe environment, the patients described how they participated in the decision making and received care without fear of being dismissed, ignored or judged. They also described how the PAM team were responsive to changing their approach by taking into account and valuing the patients' emotions. The main category is underpinned by three categories, displayed in Figure 1, which is presented below, illustrated by quotations.

### 3.1 Patients actively participated in the decision making

The patients described how they were actively invited to participate in decision making when planning their care. According to the patients, the participation in the decision making was achieved by the personnel providing them with situated knowledge and information, and actually listening to the patient's history and needs.

I was involved in all decisions made … I was also involved in the decision to go to the hospital … even though… in the beginning I did not agree to visit the PED. (Interview # 7)

The patients said that the PAM team collaborated with them, adjusting the care out of their needs. The planning of care was also carried out in collaboration with the patients' families/next of kin when they were present in the caring encounter. During the interviews, the patients talked about how their families/next of kin also received situated knowledge, information and support when it was necessary.

It was not that they [PAM] forced me… because I didn’t want to go… but then they talked to my parents and together we had a discussion and decided that the hospital was the best for me in this situation. (Interview # 4)

When there was a mutual decision not to transport the patient to the next level of care, that is the PED, the patients described how they received self‐care advice on how to handle the situation until they resumed contact with their regular caregivers or if their symptoms of illness increased or appeared again.

They told me when to take the sleeping medicine… to wait until I was in bed; in that way I would reduce the risk of falling… They also told me to contact the ordinary (psychiatric) mobile team or them (the PAM) before harming myself. (Interview # 8)

### 3.2 Patients received care without fear of being dismissed, ignored and judged

All patients stated that the PAM team provided care without dismissing, ignoring or judging them, even if the patients did not always agree on which solution would be the best for them. The patients also described their experienced feelings of frustration, anger or sadness when they realized that the PAM had arrived, especially when somebody else had made the emergency call. However, these initial feelings of frustration, anger and/or sadness mostly changed during the caring encounter, and they said that the PAM team valued their emotions and adjusted the encounter according to the patients’ frame of mind.

I have met the PAM team once before… but with other symptoms…. But this time it was a bit different… I was cheeky and a bit panicked [when the PAM arrived] … but I still thought they were doing quite well. They read well how I felt, they did not depress my mood, rather the opposite… so we could talk… (Interview # 9)

When the patients had made the emergency call themselves, they often felt relieved when the PAM arrived at the scene. However, even if they felt relieved, they also talked about a fear of meeting the personnel, not knowing who they were. But overall, they said that the caring encounter took place with ‘no drama and everything went smoothly’ since the PAM team were responsive to changing their approach and adjusting the care in the encounter according to the patients' emotions. Some patients described how they had no expectations of the care, while others expressed their wish to feel cared for by the PAM. While the patients described their experiences of the caring encounter with the PAM team, they remembered their previous experiences of being cared for by ambulance personnel, the police or other mental health‐care services.

…you can meet ambulance personnel who are professional, competent and really skilled, and next time you meet ambulance personnel that are prejudiced… they think you are stupid and treat you as such… I mean they are probably good in life‐saving situations but they are not able to talk and listen’ (Interview # 9). Neither the police nor the ambulance personnel understood me and my situation. (Interview # 5)

The patients also described how they felt humiliated, exposed and vulnerable when the police and/or ambulances arrived at their homes. They also described a wish that the PAM vehicle should be anonymous, as they did not want their neighbours to know about their illness.

The neighbours do not need to know I’m sick… (Interview # 14)

When the patients related their previous experiences of other mental health‐care services, they most commonly described a lack of trust, lack of ability and/or helplessness due to a lack of knowledge about the health‐care system.

I have bad experiences with my primary care contacts/outpatient contacts and it felt better to receive help right away. (Interview # 6)

### 3.3 The professional personnel created a safe caring environment

All the patients described how the personnel created a safe caring environment with small talk, giving sufficient time in the caring encounter, and adjusting the caring environment based on the patient's wishes. All the patients talked about the importance of small talk during the caring encounter. The small talk was described as a good distractor if they had hallucinations, for example.

Then they talked and asked general questions about anything, so that my mind was distracted… so you do not have to think about what it’s like right now… (Interview # 9)

The small talk also created a sense of trust and security and encouraged a safe place for open dialogue. The patients also described how the environment in the vehicle supported the sense of safety and also said they appreciated it when they had the chance to talk in their own homes. Several patients mentioned that it was good to have the possibility to sit and talk in the vehicle instead of lying on a stretcher.

I think it's great to sit in a chair instead of lying on a stretcher… I mean I'm not physically injured I don't need to lie down to talk… (Interview # 5)

Regardless of which environment they preferred to talk in, the most important factor was that the PAM gave the time needed and created a calming atmosphere during the situation, and the patients felt confident that the PAM team were professional, empathic and took responsibility for the situation. However, some of the patients also described how they disliked seeing personnel who had pre‐knowledge of their history.

I would have preferred it to be somebody that I did not know… I knew him from earlier… It did not feel fair that he knew me… (Interview # 6)

On the other hand, pre‐knowledge of the patient's history was experienced as something good by other patients; in these cases, the patients said that they appreciated the fact that they did not have to explain everything.

## 4 DISCUSSION

In the exploration of the patients' experiences of being cared for by the PAM team, it becomes clear that the patients were actively involved in the decision making about their care. It is likely that this participation in the decision making was possible because mutual trust and confidence were created in the caring encounter. The study also highlights how important it is for the patients not to feel dismissed, ignored or judged. It is known that participation is one way of improving the quality of health care.^26^ However, patient participation in psychiatric emergencies is not always possible and participation may not be suitable for everyone.^27^ However, by identifying factors which may affect the patients' preference for involvement, caregivers may be more sensitive to individual preferences and thus provide better person‐centred care.^28^ The concept of person‐centred care includes needs for both patients and health‐care professionals to understand and to be understood, shared decision making and the act of caring focus on the person own knowledge and experiences.^29^ As the patients addressed in this study, the PAM team created good conditions for the patients to participate in the care by being empathic, communicating in a calming way, and working to gain trust and understanding in the situation that had caused the psychiatric emergency. The PAM also invited the patients' next of kin, when present, to participate in the care. Altogether, the caring delivered by the PAM team resulted in outcomes that were attuned with the patients' needs and wants. This accords with findings reported in previous studies, 7, 28 and it shows that ‘the patient's world, vulnerability, health and suffering are primary in the art and act of caring’ p.288.^25^ However, the participants with previous experience of psychiatric emergencies compared them and reported that the act of caring was most commonly absent in a police and/or ambulance response. An interpretation of this could be that both the EMS and police departments need more knowledge about how to take care of persons in acute psychiatric distress. The need for ambulance personnel to be trained to a higher level in caring for patients facing psychiatric emergencies is known from previous studies,^5^, ^30^ and when the police work with mental health personnel, the outcome is better for the patient, the organization and the personnel.^7^, ^8^, ^10^ The findings of this study may also indicate shortcomings in the mental health‐care system; the patients described both a lack of trust and a lack of availability to their ordinary health‐care facilities; and the PAM sometimes becomes a substitute for other mental health caregivers. However, this finding may be caused by a selection bias in the participants and may also relate to a lack of knowledge about the patients' previous experiences of other mental health caregivers. But, it is reasonable to assume that the PAM is a complement for handling psychiatric emergencies in the EMS, since the police and ambulance services have limited resources and sometimes sparse knowledge of caring for patients with such emergencies. The participants described how they sometimes felt humiliated, exposed and vulnerable when the police and/or ambulances arrived at their homes, and this finding could be connected with the stigmatization of mental illness. Stigmatization may be seen as negative for the individual and is often associated with rejection and being assessed as ‘not normal’ by the surrounding community.^31^ From the study findings, it is clear that the PAM team took part in the patients' lived experience and shared their professional knowledge, but a paradox appeared when the team arrived in a vehicle resembling as a PAM ambulance. The patients wanted them to arrive in silence; they did not want their neighbours to know about their illness. How to achieve the balance between avoiding public scrutiny and ensuring a rapid response is an issue for further consideration, as highlighted and also discussed in a previous study.^7^

### 4.1 Methodological considerations

To facilitate credibility, data analysis was initially performed by two of the authors, and the other author confirmed the analysis. During the analysis, numerous discussions took place until consensus was achieved. To further strengthen credibility, quotations were used to illustrate the results. The pre‐understanding among the researchers can be considered as a potential limitation since there may be barriers to openness when analysing the data. However, to visualize and handle our pre‐understanding and reduce the risk of influence the research process, we had critical reflections and discussions during the whole process. To facilitate dependability, the majority of interviews were conducted by one of the authors using an interview guide. It may seem remarkable that all patients had a positive experiences with the PAM; this may be caused by the semi‐structured questions used; and implicitly, a caring encounter can be perceived as something positive. There is also a possibility that the patient wanted to give the ‘right’ answers since there always is a risk of non‐equality between researchers and participants. The positive results may also be caused by a selection bias due to the convenience data collection. However, there is no way of evaluating the selection bias due to lack of knowledge about eligible patients not participating in the study. The majority of interviews were conducted by telephone, and this may have led to lack of detection of nuances of the patients' experiences; however, the telephone enabled the inclusion of 13 participants who might otherwise have been excluded due to their unwillingness to meet face‐to face. Despite the possible disadvantages of using telephone interviews for data collection, it has been shown that qualitative telephone interviews represent a valuable technique for data collection in sensitive topics.^32^ In this study, participants were asked about their willingness to participate at the end of the caring encounter by the RNs, and there may have been a risk that the RNs only asked patients who were satisfied with the care delivered. Another selection bias is that when the researchers tried to establish contact with the patients, only 53% were reachable after three phone calls and one or two text messages. The inclusion of non–native‐speaking participants may also have been a limitation. However, this was not perceived as an issue by the interviewers, and by including those participants, greater variation among the participants was achieved. The participants may not have been representative of all patients cared for by the PAM but the findings reflect their perspective and correspond to those in a similar study.^8^ In qualitative studies, the aim is not to identify representative participants but to describe the patients' experiences. Therefore, the findings of this study can be seen as a part of a picture, and additional studies are needed to further explore these experiences.

### 4.2 Relevance for clinical practice

Since psychiatric emergencies are increasing in the EMS, there is a need for specialist response units such as the PAM in the EMS. The alternative to use of specialist response units is to increase the accessibility to mental health‐care services outside office hours and to increase knowledge among police and EMS personnel about handling psychiatric emergencies. However, the best situation for the patients might be the use of specialist response units such as the PAM as a part of the EMS, in collaboration with the police department and the ambulance service. This collaboration between different organizations could lead to a transition of knowledge, and this increased knowledge could result in patients being invited to participate in their care also by the police and ambulance service. Due to the patients' expressed fear of stigmatization about mental illness, the specialist response units might consider using a civil vehicle which has no resemblance to a PAM ambulance.

## 5 CONCLUSION

The patients' experiences of being cared for by the PAM team show that person‐centred care was achieved by involving the patients in the care, and this participation was possible because mutual trust and confidence were established. Having a profound knowledge of the psychiatric patient's vulnerability seems to be a prerequisite for being able to provide person‐centred care. The patients acknowledged a specialist response unit to be a valuable part of the EMS, especially when they described their perceived lack of accessibility to other health‐care facilities. However, further studies are needed to explore whether the PAM as a response unit in the EMS decreases the risk of suicide and to examine different health economic aspects of using PAM in the EMS.

## CONFLICT OF INTEREST

All the authors declare that they have no conflict of Interest.

## ETHICAL APPROVAL

Care seekers cared for by the PAM team in a psychiatric emergency are a vulnerable group of individuals. They or their relatives have sought help for an acute illness, and the care seekers could be considered as dependent in relation to the specialist nurse in PAM. For this reason, a first request for participation in the study was made by the specialist nurse in PAM when the need for care had been assessed and at the end of the caring encounter. If the patient expressed interest in participation in the study, they received written information about the study, including matters of anonymity, integrity and confidentiality. The patients were only invited to participate if their contact details were known, they could converse in Swedish, and they were assessed by the specialized nurse as being sufficiently well to understand the information and the question about participation in the study. The researchers contacting the eligible participants checked whether they had understood the written information before consent to participate was requested. Consent to participate was given by all participants, either written or orally (audio‐recorded). The participants were informed that they could retract their agreement to participate at any time without any consequences. The study was approved by the Ethical Review Board in Stockholm, Sweden (2014/1816‐31/5 & 2018/103‐22). Furthermore, the study was designed to comply with the ethical principles of research described by the International Council of Nurses, which require researchers to ensure the anonymity, integrity and confidentiality of the participants.

## Data Availability

The data used and analysed during the current study are available from the corresponding author on reasonable request.
